# Dietary and Lifestyle Patterns and Their Associations with Cardiovascular and Inflammatory Biomarkers in Vegans, Vegetarians, Pescatarians, and Omnivores: A Cross-Sectional Study

**DOI:** 10.3390/nu17233634

**Published:** 2025-11-21

**Authors:** Izabela Kwiatkowska, Jakub Olszak, Dorota Formanowicz

**Affiliations:** 1 Chair and Department of Medical Chemistry and Laboratory Medicine, Poznan University of Medical Sciences, 61-701 Poznan, Poland; ikwiatkowska@ump.edu.pl; 2 Institute of Computing Science, Poznan University of Technology, 60-965 Poznan, Poland; jakub.olszak@cs.put.poznan.pl

**Keywords:** plant-based diets, lifestyle factors, body composition, lipid profile, inflammatory markers, apolipoproteins, cardiovascular risk

## Abstract

**Background:** Plant-based diets are associated with reduced cardiometabolic risk, yet the influence of lifestyle behaviors on these benefits remains insufficiently understood. **Objective:** To assess the combined impact of dietary patterns and lifestyle behaviors on body composition, lipid profiles, and inflammatory biomarkers in healthy young adults. **Methods:** In this cross-sectional study, 155 participants aged 18–39 years were categorized into four dietary groups: vegans (*n* = 48), vegetarians (*n* = 49), pescatarians (*n* = 23), and omnivores (*n* = 35). Body composition was measured using bioelectrical impedance analysis. Blood samples were analyzed for lipid parameters, apolipoproteins, lipoprotein(a), and inflammatory markers (IL-6, TNF-α, and hsCRP). Participants were further stratified based on behavioral factors, including physical activity, sleep duration, smoking, and alcohol consumption. **Results:** Vegans demonstrated the lowest body fat and visceral adipose tissue, along with the second-highest skeletal muscle mass. Significant intergroup differences were observed in total cholesterol (*p* = 0.032), HDL-C (*p* = 0.006), and triacylglycerols (*p* = 0.005). Among vegans, suboptimal lifestyle behaviors were associated with elevated LDL-C, non-HDL-C, and homocysteine levels (*p* < 0.05). Positive correlations were identified between ApoB and BMI (r = 0.517) and between IL-6 and waist–to–hip ratio (ρ = 0.499). **Conclusions:** A vegan diet, when combined with healthy lifestyle behaviors, is associated with favorable body composition and lipid profiles. Regardless of dietary pattern, maintaining a healthy body weight and minimizing visceral adiposity are essential for reducing cardiovascular and inflammatory risk. These research findings underscore the importance of integrating high-quality plant-based diets with lifestyle modifications and advanced modeling approaches.

## 1. Introduction

In the 21st century, individuals are increasingly exposed to a fast-paced lifestyle and chronic stress, both of which pose significant challenges to maintaining optimal health. In recent years, scientific discourse has emphasized six foundational pillars of health: nutrition, physical activity, stress management, restorative sleep, social connection, and avoidance of harmful substances [1]. These lifestyle components exert a profound influence on overall health status and are critical determinants of disease risk and longevity [2,3,4].

Non-communicable diseases (NCDs)—including obesity, type 2 diabetes, cardiovascular diseases, cancer, and metabolic syndrome—are closely linked to chronic low-grade inflammation. This form of inflammation is often asymptomatic and may progress silently, contributing to systemic damage even when conventional inflammatory markers remain within normal limits. Over time, persistent inflammation can lead to serious health consequences, including neuroinflammation, which is implicated in cognitive decline and neurodegenerative disorders such as dementia and Alzheimer’s disease. Inflammatory processes are also involved in the pathogenesis of conditions such as rheumatoid arthritis and endometriosis. Elevated inflammatory markers have been observed in individuals with depression, prompting growing interest in dietary strategies aimed at mitigating inflammation [5,6,7,8,9,10,11,12,13,14,15,16,17,18].

Nutritional factors are increasingly recognized as key modulators of systemic inflammation. Diets rich in high-quality, anti-inflammatory nutrients have been shown to significantly reduce circulating inflammatory markers [17,19,20]. Anti-inflammatory dietary patterns aim to attenuate inflammation associated with lifestyle-related risk factors. These diets emphasize the consumption of fruits, vegetables, legumes, nuts, seeds, whole grains, and culinary herbs and spices, while ensuring adequate intake of high-quality proteins and unsaturated fats. While the Mediterranean diet remains the most extensively studied, other anti-inflammatory dietary models include vegetarian, pescatarian, vegan, and planetary diets [5,21,22,23,24,25].

Epidemiological studies consistently demonstrate that dietary patterns characterized by high intake of plant-based foods and/or regular fish consumption are associated with lower levels of systemic inflammation [24]. In contrast, diets high in saturated fats and low in dietary fiber are linked to pro-inflammatory responses, including elevated levels of C-reactive protein (CRP) and interleukin-6 (IL-6) [25]. Furthermore, plant-based diets are rich in bioactive compounds, such as polyphenols and antioxidants, that help modulate inflammatory pathways. This process may result in decreased levels of CRP and IL-6 in the bloodstream.

Recent updates to global dietary guidelines increasingly promote plant-based eating patterns [26,27,28,29,30]. The 2019 EAT–Lancet Commission report advocates for a planetary health diet, emphasizing increased consumption of plant-based foods—particularly fruits, vegetables, legumes, whole grains, and nuts—while reducing intake of animal-derived products. This dietary shift is proposed to improve both human health and environmental sustainability [31].

According to the latest data from the Proceedings of the Nutrition Society, the term “plant-based diet” encompasses a broad spectrum of dietary patterns that prioritize plant-derived foods and limit or exclude animal products [32]. These include various vegetarian diets, the portfolio diet, the planetary diet, the Mediterranean diet, and the Dietary Approaches to Stop Hypertension (DASH) diet. The portfolio diet, primarily a vegetarian approach, emphasizes daily intake of 42 g of nuts, 50 g of plant-based protein, 20 g of soluble fiber, and 2 g of plant sterols. The Mediterranean diet allows for moderate consumption of low-fat dairy and white meat, includes oily fish as a source of healthy fats, and promotes the consumption of plant-based foods such as olives, legumes, and extra-virgin olive oil [33]. The DASH diet, a variant of the Mediterranean model, focuses on reducing sodium, added sugars, and saturated fats, while promoting fruits, vegetables, legumes, whole grains, lean proteins, and low-fat dairy [34].

The Mediterranean, DASH, and WHO dietary guidelines consistently recommend replacing red and processed meats with alternative protein sources such as fish, legumes, eggs, and nuts. These patterns also emphasize minimizing the intake of ultra-processed foods [27,31,33,34].

Within vegetarian dietary classifications, “vegetarian” typically refers to individuals who exclude meat and fish but may consume eggs and dairy products. Subtypes include lacto-vegetarians, ovo-vegetarians, and lacto-ovo vegetarians. Pescatarians, a less restrictive group, include fish and seafood in their diets alongside eggs and dairy. Flexitarians, or semi-vegetarians, primarily follow a plant-based diet but occasionally consume meat [30,35]. In contrast, vegan diets exclude all animal-derived products, including meat, fish, dairy, eggs, and by-products such as gelatin and honey [35,36].

Several subtypes of vegan diets have been described in the literature. These include the recommended whole-food vegan diet, which avoids processed foods, and the low-fat whole-food vegan diet, which limits high-fat plant-based items. Conversely, the raw vegan diet excludes all cooked or heat-processed foods and lacks robust scientific validation [35,37,38].

**Figure 1:**
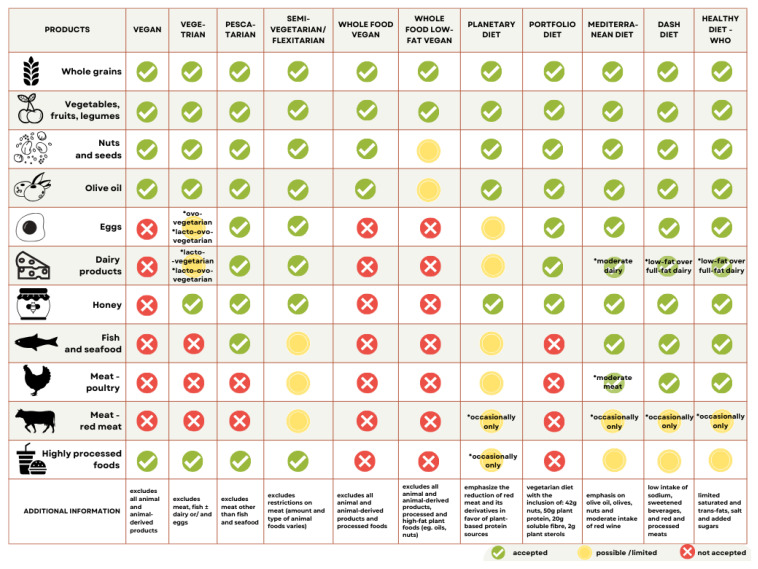
Anti-inflammatory and plant-based diets and their characteristics. *—contains additional information.

Figure 1 illustrates the characteristics of the analyzed dietary patterns in comparison to the WHO recommendations for a healthy diet [26,30,35,36,39].

An expanding body of evidence supports the health benefits of vegetarian and vegan diets, particularly in the prevention of chronic diseases, enhancement of overall health, and reduction in environmental impact [35,40,41,42,43,44,45,46]. Individuals adhering to plant-based diets exhibit a lower prevalence of cardiovascular disease, certain types of cancer, type 2 diabetes, hypertension, and obesity. Moreover, modifiable cardiovascular risk factors—such as excess body weight and lipid profile parameters, including total cholesterol (TC), low-density lipoprotein cholesterol (LDL-C), and apolipoprotein B—tend to be more favorable among vegetarians, especially vegans, compared to omnivores. Plant-based diets are also increasingly recognized as effective components of therapeutic interventions [45,47,48,49,50,51,52,53,54,55,56,57]. Despite their potential health benefits, vegetarian and vegan diets remain subjects of debate in both public and scientific discourse. The health-promoting effects of these diets depend on their nutritional adequacy. Poorly planned plant-based diets, particularly those that exclude key food groups without suitable substitutes, may lead to nutrient deficiencies in essential nutrients such as vitamin B12, iron, and zinc, and diminish the potential health benefits [35,58]. The present study aims to investigate how dietary patterns and lifestyle behaviors among vegans, vegetarians, pescatarians, and omnivores influence cardiovascular and inflammatory biomarkers, with a particular focus on body composition and modifiable risk factors for cardiovascular disease.

## 2. Materials and Methods

### 2.1. Study Design

This cross-sectional study was conducted between 24 June and 13 July 2021, at the Department of Medical Chemistry and Laboratory Medicine, Poznan University of Medical Sciences. The study adhered to the principles of the Declaration of Helsinki and received approval from the Bioethics Committee of the Poznan University of Medical Sciences (Approval No. 237/20/2020). All participants provided written informed consent before enrollment.

Participants were recruited via online platforms, including social media and targeted email invitations. To ensure adequate representation of plant-based dietary groups, recruitment was extended to closed online communities focused on vegetarian and vegan lifestyles in Poland, including “Vegetarians and Vegans” (with over 24,000 members) and “Vegans Poland” (with 59,000 members). Eligible individuals were invited to attend the study site at a scheduled time. Before participation, they received detailed instructions regarding inclusion and exclusion criteria, fasting requirements, and contraindications.

The study was conducted in two phases. In the first phase, participants underwent a body composition assessment using bioelectrical impedance analysis (BIA) and completed a Food Frequency Questionnaire (FFQ) to evaluate their dietary habits. Detailed quantitative analysis of the FFQ data, including calculation methods and validation procedures, has been presented in our previous publication [47]. Behavioral factors, including physical activity, smoking status, alcohol consumption, and sleep duration, were evaluated using a brief, structured questionnaire administered on-site. The findings from this phase have been previously published [47].

Participants who met the inclusion criteria and had normal results in basic laboratory tests (lipid profile and fasting glucose) were invited to participate in the second phase of the study. This phase included advanced biochemical analyses (with new requirements for inclusion in the study) and a follow-up analysis of body composition involving a select group of participants from the initial stage who also met additional exclusion criteria. The current manuscript focuses on the results of this second phase.

**Figure 2:**
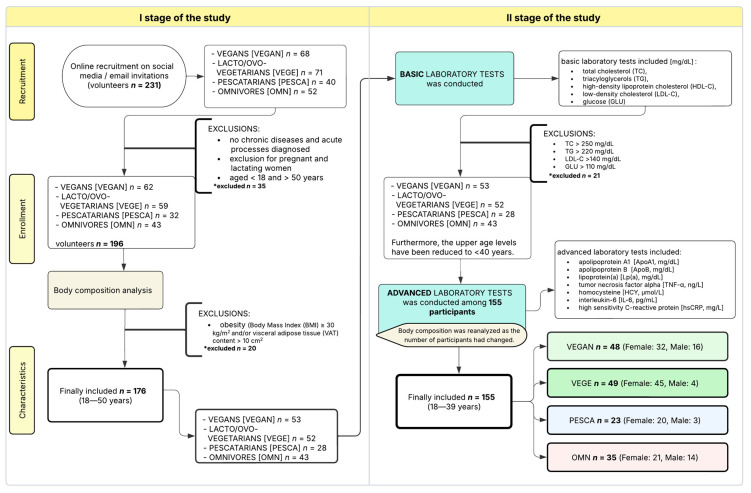
The scheme of recruiting participants.

The recruitment process is illustrated in Figure 2.

### 2.2. Subjects

The study population consisted of healthy adults aged 18–39 years who adhered to one of the following self-reported dietary patterns:

- **Vegan diet (VEGAN):** excludes all animal-derived products;
- **Lacto-/ovo-vegetarian diet (VEGE):** includes dairy and/or eggs, excludes meat and fish;
- **Pescatarian diet (PESCA):** includes fish, eggs, and dairy products;
- **Omnivore/mixed/traditional diet (OMN):** regular consumption of meat.

Dietary adherence was confirmed through self-report and verified using the FFQ administered during the first phase [47]. Participants were required to have followed their declared dietary pattern for at least one year.

Exclusion criteria included pregnancy or lactation, presence of chronic or acute illnesses, and age outside the specified range. Of the 196 individuals initially recruited, 176 completed the first phase. Twenty participants were excluded due to obesity identified via body composition analysis. In the second phase, an additional 21 participants were excluded due to abnormal laboratory values (e.g., TC ≥ 250 mg/dL, triacylglycerols (TG) ≥ 220 mg/dL, LDL-C ≥ 140 mg/dL, fasting glucose (GLU) ≥ 110 mg/dL). Ultimately, 155 participants were included in the final analysis.

### 2.3. Body Composition and Behavioral Factors Questionnaire Analysis

Body composition was assessed using the InBody 120 analyzer (InBody Co., Seoul, Republic of Korea), which employs Direct Segmental Multi-frequency Bioelectrical Impedance Analysis (DSM-BIA). The following parameters were measured: body weight (kg), skeletal muscle mass (SMM, kg), total body fat mass (BFM, kg and %), visceral adipose tissue (VAT, cm^2^), total body water (L), protein (kg), minerals (kg), body mass index (BMI, kg/m^2^), percentage body fat (PBF), and waist–hip ratio (WHR).

Participants were instructed to fast for 14–16 h, avoid fluid intake for at least 1 h before the test, and refrain from intense physical activity for 24 h preceding the test. Contraindications included pregnancy and the presence of implanted electronic devices (e.g., pacemakers). No participants reported contraindications.

Behavioral factors were assessed using a structured questionnaire administered on the day of testing. The questionnaire assessed physical activity (in minutes/week), sleep duration (in hours/night), smoking status, and alcohol consumption. In line with the core pillars of Lifestyle Medicine, a minimum of 150 min of physical activity per week is recommended, along with sufficient sleep duration of around 7 h per night. Moreover, individuals are advised to abstain from risky substances, particularly tobacco and alcohol [1]. Alcohol consumption was evaluated based on self-reported types and frequency of intake (from FFQ). The analysis focused exclusively on vodka as the primary high-strength alcohol, given its prevalence in the studied population. Wine and beer were excluded because of inconsistent evidence regarding their health effects, particularly the potential protective role of moderate wine consumption.

Based on responses, participants were categorized into subgroups reflecting appropriate (ABF) or inappropriate (IBF) behavioral factors, as described in Section 2.5.

### 2.4. Biochemical Tests

Venous blood samples were collected after 14–16 h of fasting. Basic biochemical parameters—including TC, TG, high-density lipoprotein cholesterol (HDL-C), LDL-C, and fasting GLU—were measured using the Cobas b 101 analyzer (Roche Diagnostics) and COBAS INTEGRA 400 plus system.

Participants with values exceeding predefined thresholds (TC ≥ 250 mg/dL, TG ≥ 220 mg/dL, LDL-C ≥ 140 mg/dL, GLU ≥ 110 mg/dL) were excluded from further testing.

Advanced biochemical analyses included apolipoprotein A1 (ApoA1), apolipoprotein B (ApoB), lipoprotein(a) [Lp(a)], tumor necrosis factor-alpha (TNF-α), homocysteine (HCY), interleukin-6 (IL-6), and high-sensitivity C-reactive protein (hsCRP). These were measured using enzyme-linked immunosorbent assay (ELISA) kits (SunRed and DRG Diagnostics) and analyzed using the TECAN SUNRISE reader with Magellan v3.0 software.

Derived lipid ratios included ApoB/ApoA1, TC/HDL-C, and non-HDL-C. Confidence intervals (CI) for selected parameters (ApoA1, ApoB, TNF-α, IL-6, and hsCRP) were calculated and are presented in Supplementary Table S1.

### 2.5. Processing of Data—Subgroups

Participants in each dietary group were stratified into two behavioral subgroups:

- **Appropriate Behavioral Factors (ABF):** score > 3 points
- **Inappropriate Behavioral Factors (IBF):** score ≤ 3 points

Scoring was based on four lifestyle domains: physical activity, sleep duration, smoking, and alcohol consumption. Each domain contributed a maximum of one point, with higher scores indicating healthier behaviors. Each behavioral factor was scored according to adherence to healthy lifestyle recommendations. A maximum of 1 point was assigned for the most appropriate behavior, 0.5 points for a moderately appropriate response, and 0 points for inappropriate behavior in each category. The criteria were based on the Lifestyle Medicine recommendations [1].

The scoring criteria are detailed in Section 3, Table 1.

### 2.6. Statistical Analysis

Statistical analyses were performed using Jamovi statistical software (version 2.3) [59]. The Kruskal–Wallis one-way ANOVA test was used to assess differences among the four study groups. Significant Kruskal–Wallis findings were followed by Dwass-Steel-Critchlow-Fligner (DSCF) post hoc pairwise comparisons. Comparisons between two independent groups (ABF and IBF) were performed using parametric or nonparametric tests, depending on data distribution and homogeneity of variances. Normality was evaluated using the Shapiro–Wilk test, and homogeneity of variances using Levene’s test. Statistical significance was set at *p* < 0.05.

Pearson correlation coefficient (r) was used to assess relationships between normally distributed variables, whereas Spearman’s rank correlation coefficient (ρ) was applied for non-normally distributed data.

## 3. Results

The study was conducted at the Department of Medical Chemistry and Laboratory Medicine, Poznan University of Medical Sciences, in Poland. It included healthy individuals adhering to one of four dietary patterns: vegan (VEGAN), lacto-/ovo-vegetarian (VEGE), pescatarian (PESCA), and traditional omnivorous diet (OMN).

Participants in the VEGAN, VEGE, and PESCA groups had followed their respective diets for an average of 4.0, 7.9, and 4.4 years, respectively. Notably, many individuals in the VEGAN group had previously followed a lacto-/ovo-vegetarian diet, suggesting a longer overall duration of meat exclusion.

### 3.1. Baseline Characteristic and Behavioral Factors Outcomes

The study included 155 participants, predominantly female (see Figure 2 in Section 2). All individuals were under the age of 40. The mean ages in the OMN, PESCA, VEGE, and VEGAN groups were 30.0 ± 3.85, 29.3 ± 6.46, 28.2 ± 5.95, and 29.6 ± 6.35 years, respectively. Although age was not controlled during recruitment, no statistically significant differences were observed among the groups.

Responses to the lifestyle questionnaire were similar across all dietary groups, indicating average levels of physical activity, sleep duration of 7–8 h, and low prevalence of smoking and alcohol consumption. No statistically significant differences were found in these behavioral factors. Table 1 presents the baseline characteristics and behavioral outcomes.

Analysis of the data revealed that the VEGAN group had the highest percentage of individuals who abstained from smoking (83.3%) and alcohol consumption (72.9%). However, nearly 20% of this group reported low physical activity. Additionally, only 6.3% reported insufficient sleep duration (≤6 h), while 14.6% reported sleeping ≥9 h—more than in other groups.

The VEGE group had the highest proportion of participants reporting inadequate sleep (26.5%) and the lowest percentage reporting optimal sleep duration (6–8 h).

The PESCA group showed the highest prevalence of smoking, with nearly 35% reporting occasional or regular use.

The OMN group was the most physically active, with almost 30% reporting high activity levels. However, only 48.6% of this group reported abstaining from vodka consumption—the lowest among all groups.

Table 2 shows the distribution of participants into behavioral subgroups. The ABF subgroup (score > 3) included 52.08% of vegans—the highest proportion among all groups. Pescatarians followed with 39.13%. In contrast, only ~32% of vegetarians and omnivores were classified as ABF. Over 60% of participants in the VEGE, PESCA, and OMN groups were assigned to the IBF subgroup.

Further statistical comparisons were conducted between subgroups with appropriate (ABF) and inappropriate (IBF) behavioral factors within the VEGAN group. This was due to the sufficient sample size and balanced distribution between ABF and IBF in this group. The results of these subgroup analyses are presented in the tables in Section 3.3.

### 3.2. Body Composition Analysis

Body composition parameters were assessed using the InBody 120 analyzer, which applies Direct Segmental Multi-frequency Bioelectrical Impedance Analysis (DSM-BIA). The results are summarized in Table 3.

No statistically significant differences were observed between ABF and IBF subgroups; therefore, subgroup data are not presented in this section.

Significant differences were found among dietary groups for body water (*p* = 0.016), protein (*p* = 0.015), minerals (*p* = 0.033), and skeletal muscle mass (SMM; *p* = 0.014). DSCF post hoc comparisons (Supplementary Table S2) revealed significant differences between VEGAN and VEGE groups in all cases. The OMN group exhibited the highest values for these parameters, while the VEGE group showed the lowest.

The OMN group also had the highest values for body fat mass (BFM), body weight, body mass index (BMI), waist–hip ratio (WHR), and visceral adipose tissue (VAT). In contrast, the VEGE group had the lowest body weight and WHR, but the highest percentage body fat (PBF).

The PESCA group demonstrated the lowest BFM and BMI values among all groups.

The VEGAN group showed the lowest levels of total and visceral fat tissue. Their muscle mass, protein content, body water, and mineral levels were the second highest, comparable to those of omnivores, but distinct from the VEGE and PESCA groups.

### 3.3. Biochemical Blood Tests

Biochemical blood test results—including lipid profiles, apolipoproteins, lipoprotein(a), and inflammatory markers—are presented in Table 4, Table 5, Table 6 and Table 7.

All measured parameters remained within recommended reference ranges [60,61,62,63,64,65,66].

Statistically significant differences among dietary groups were observed for: TC (*p* = 0.032); HDL-C (*p* = 0.006); TG (*p* = 0.005). No significant differences were found for LDL-C, non-HDL-C, ApoA1, ApoB, or lipoprotein(a) when comparing the four dietary groups. Post hoc DSCF pairwise comparisons (Supplementary Table S3) indicated that for TG, the OMN group differed significantly from both the VEGAN and VEGE groups (*p* < 0.05). Additionally, for HDL-C, the VEGAN group showed a significant difference from the VEGE group (*p* < 0.05).

Subgroup analysis based on behavioral factors (ABF vs. IBF) was feasible only within the VEGAN group due to sufficient sample size and balanced distribution.

As shown in Table 6, the IBF subgroup exhibited significantly higher values of: Lp(a) (*p* = 0.029); LDL-C (*p* = 0.005); non-HDL-C (*p* = 0.039).

ApoB levels were also higher in the IBF subgroup, approaching statistical significance (*p* = 0.050).

When comparing all participants classified into ABF and IBF subgroups (regardless of diet), significant differences were found in: Lp(a) (*p* = 0.042); LDL-C (*p* = 0.024).

Metabolic and inflammatory markers—including homocysteine (HCY), TNF-alpha, IL-6, and hsCRP—did not differ significantly among dietary groups (see Table 7).

However, within the VEGAN group, the IBF subgroup showed a significantly higher homocysteine concentration compared to the ABF subgroup (*p* = 0.007), suggesting a potential link between lifestyle factors and cardiovascular risk.

**Figure 3:**
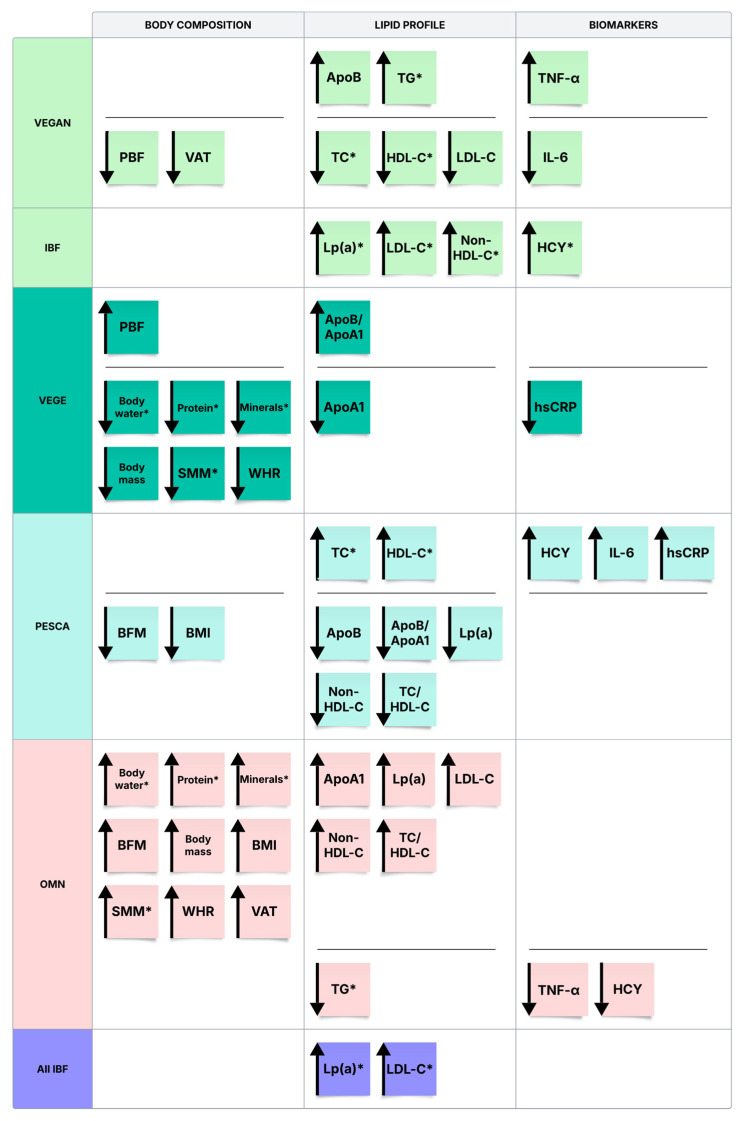
Summary of Key Biochemical and Anthropometric Findings Across Dietary and Behavioral Subgroups. Abbreviations: ApoA1—Apolipoprotein A1; ApoB—Apolipoprotein B; ApoB/ApoA1—apolipoprotein B/apolipoprotein A1 ratio; HCY—Homocysteine; PBF—Percentage Body Fat; BFM—Body Fat Mass; BMI—Body Mass Index; HDL-C—High-density lipoprotein cholesterol; hsCRP—High sensitivity C-reactive protein; IL-6—Interleukin 6; LDL-C—Low-density lipoprotein cholesterol; Lp(a)—Lipoprotein(a); Non-HDL-C—Non-high-density lipoprotein cholesterol; TC—Total cholesterol; TC/HDL-C—Total cholesterol to high-density lipoprotein cholesterol ratio; TG—triacylglycerols; TNF-α—Tumor Necrosis Factor-alpha; SMM—Skeletal Muscle Mass; WHR—Waist-Hip Ratio; VAT—Visceral Adipose Tissue; ↑—The highest average value among the groups; ↓—The lowest average value among the groups.

Key findings are visualized in Figure 3, which highlights the highest and lowest average values across dietary and behavioral subgroups. Statistically significant results are marked with an “*”.

### 3.4. Correlations Between the Biochemical Analyses and Body Composition Parameters in Subgroups

Correlation analyses were conducted across all dietary and behavioral subgroups. However, statistically significant associations were observed exclusively within the VEGAN-ABF subgroup (vegans with appropriate behavioral factors). These findings are summarized in Table 8 and detailed in Supplementary Table S1.

In the VEGAN-ABF subgroup, several moderate to strong positive correlations were identified between lipid and inflammatory biomarkers and body composition metrics. ApoB and BMI showed a strong positive correlation, suggesting that increased body mass is associated with elevated levels of atherogenic lipoproteins. IL-6 was positively correlated with body mass and WHR, indicating that abdominal fat distribution may contribute to systemic inflammation, even among individuals adhering to a vegan diet and healthy lifestyle behaviors.

These findings underscore the importance of maintaining optimal body composition—particularly minimizing visceral adiposity—as a key factor in reducing cardiovascular and inflammatory risk, regardless of dietary pattern.

## 4. Discussion

### 4.1. Body Composition and Lifestyle Characteristics Across Dietary Groups

The body composition analysis revealed that the VEGAN group exhibited several favorable physiological traits. This group demonstrated the second-highest skeletal muscle mass, comparable to the OMN group, and exceeding that of VEGE and PESCA by 3 and 2.5 percentage points, respectively. This finding contrasts with earlier studies, which often reported lower muscle mass among vegans [67]. However, recent evidence by Monteyne et al. (2023) suggests that high-protein vegan diets (~2 g/kg body mass) can support muscle protein synthesis rates comparable to those of omnivorous diets [68].

In terms of body water, protein, and mineral content, the VEGAN group also performed well, ranking second after the OMN group. These parameters are closely linked to muscle mass and are crucial for bone health (as “minerals” reflect the content of minerals in bones and teeth, connected with the risk of osteoporosis or osteopenia in individuals) [69]. Although vegan diets have been associated with lower bone mineral density [70,71,72], the findings suggest that well-planned vegan diets may mitigate these risks. This is supported by studies showing no significant differences in body composition between dietary groups [73].

Importantly, the VEGAN group had the lowest total and visceral fat levels, which are key predictors of cardiometabolic risk [74,75,76,77]. These results align with large-scale data from the UK Biobank [70] and a recent meta-analysis of 13 studies, which demonstrated that predominantly plant-based diets (PPBDs) are associated with reduced visceral adiposity [77].

Despite having the highest muscle, water, protein, and mineral content, the OMN group showed the highest values for body mass, fat mass (including VAT), BMI, and WHR. In contrast, the VEGE and PESCA groups presented intermediate profiles, characterized by lower muscle mass and reduced content of water, minerals, and protein. It is essential to note that body water and protein levels are closely linked to muscle content; water is predominantly stored in muscles, while protein serves as a fundamental building block. Consequently, lower muscle mass was associated with decreased values in these two parameters. Improving these metrics may include increasing protein and calcium intake, supplementing vitamin D, and promoting physical activity [78,79,80,81,82].

Behavioral analysis further highlighted the VEGAN group’s health-conscious lifestyle. This group had the lowest prevalence of smoking (83.3%) and alcohol consumption (72.9%), and the highest proportion of individuals with adequate sleep duration. However, a notable limitation was the high percentage of participants with low physical activity (18.8%), which may influence specific metabolic outcomes discussed in later sections.

### 4.2. Connection Between Lifestyle and Lipid Panel Values

#### 4.2.1. Lipid Profile

Statistically significant differences were observed in lipid profile parameters across dietary groups. The VEGAN group exhibited the lowest levels of TC and HDL-C, while the PESCA group showed the highest levels of HDL-C and TC. Conversely, TG levels were highest in the VEGAN group and lowest in the OMN group (the VEGE group also differed significantly from OMN in post hoc comparisons). All values remained within reference ranges.

The low TC levels in the VEGAN group are consistent with the characteristics of a vegan diet, which typically includes low intake of cholesterol, trans fats, and saturated fats, and high intake of fiber and plant sterols—factors known to influence lipid levels according to ESC/EAS guidelines [64].

The elevated HDL-C in the PESCA group likely reflects the regular consumption of omega-3-rich fish, which is known to positively affect HDL-C concentrations [83,84]. This group also had the second-highest proportion of individuals with appropriate behavioral factors (ABF) and the lowest average body fat and BMI, suggesting that the higher TC may be driven by beneficial HDL-C rather than atherogenic fractions.

Elevated levels of HDL-C in vegetarians compared to vegans (Supplementary Table S3) may be attributable to distinct dietary patterns. Specifically, vegetarians often exhibit a higher intake of lipids derived from dairy and eggs, along with increased consumption of monounsaturated fats, and a reduced overall intake of fiber. These dietary factors are recognized for their beneficial effects on HDL metabolism [64].

Despite remaining within normal limits, the unexpectedly high TG levels in the VEGAN group may be attributed to lower physical activity and potential deficiencies in omega-3 intake or supplementation. Further investigation into supplementation habits is warranted. Importantly, the VEGAN participants did not report excessive intake of refined carbohydrates; their diets were rich in high-quality plant proteins such as tofu, tempeh, legumes, nuts, and seeds [47].

These findings align with meta-analyses indicating that vegan diets are associated with lower TC and HDL-C, but not necessarily with reduced TG levels [85]. Other studies present mixed results: some report no significant differences in TG between vegans, vegetarians, and omnivores [86,87], while others show lower TG in vegans compared to vegetarians [88,89].

#### 4.2.2. Apolipoproteins and Lipoprotein(a)

No statistically significant differences were observed across dietary groups in ApoA1, ApoB, or Lp(a) levels. However, subgroup analysis revealed that individuals with IBF had significantly higher levels of Lp(a), LDL-C, and non-HDL-C within the VEGAN group. ApoB levels also trended higher in this subgroup (*p* = 0.050).

These findings suggest that unhealthy lifestyle behaviors may exacerbate lipid-related cardiovascular risk, even among individuals following a vegan diet. LDL-C is known to be sensitive to lifestyle factors similar to those affecting TC. The elevated Lp(a) levels in the IBF subgroup raise questions about the dietary modulation of genetically influenced lipoproteins [90]. The findings of these studies have been characterized as both ambiguous and minor [91].

Although Lp(a) is largely genetically determined, emerging evidence suggests that diet quality may influence its concentration. A recent interventional study reported a significant reduction in Lp(a) levels following a vegan diet over a four-week period [48]. Diets rich in unsaturated fatty acids (PUFAs and MUFAs) may lower Lp(a) levels, as supported by the low values observed in the PESCA group, which regularly consumes fish and seafood [92,93].

Interestingly, a 2024 meta-analysis found that reducing saturated fat intake may paradoxically increase Lp(a), particularly when it is replaced with carbohydrates or trans fats [94]. Supplementation with flaxseed has also been shown to reduce Lp(a) levels [95], highlighting the potential role of dietary interventions in managing this lipoprotein.

### 4.3. Connection Between Lifestyle, Body Composition, and Metabolic and Inflammatory Markers

This study evaluated various biomarkers, including homocysteine as a metabolic risk factor, hsCRP as a marker of low-grade inflammation, and TNF-α and IL-6 as pro-inflammatory cytokines.

#### 4.3.1. Homocysteine (HCY)

Although no statistically significant differences in metabolic and inflammatory markers were observed across dietary groups, subgroup analysis within the VEGAN group revealed that individuals with IBF had significantly higher HCY levels compared to those with appropriate behavioral factors (ABF). This supports the hypothesis that unhealthy lifestyle behaviors, such as low physical activity and potential vitamin B deficiencies, may elevate HCY concentrations—a recognized cardiovascular risk factor [96,97,98].

The VEGAN group had the lowest physical activity levels, with most IBF participants falling into this category. Previous studies have shown that insufficient physical activity can contribute to elevated HCY levels [99,100]. Although concerns regarding vitamin B12 deficiency in vegans are well-documented [96,101,102,103,104,105,106], the average HCY levels in this study remained within normal ranges, suggesting that participants likely supplemented appropriately.

Interestingly, the PESCA group exhibited the highest average HCY concentration. This may be linked to their higher smoking prevalence and greater body fat, including visceral adipose tissue. Literature indicates that visceral fat accumulation is associated with increased HCY levels, warranting further investigation [107,108,109].

#### 4.3.2. TNF-α, IL-6, hsCRP

Research comparing inflammatory markers across dietary patterns remains limited, particularly regarding pescatarians. Many studies either exclude this group or aggregate vegetarians without distinguishing between subtypes. This highlights the need for more nuanced analyses.

A recent study comparing hsCRP, IL-6, and TNF-α among vegans, vegetarians, and omnivores found that TNF-α levels were lowest in omnivores, consistent with our findings for the OMN group. Similarly, hsCRP concentrations were highest in omnivores and lowest in vegans, aligning with our results. However, IL-6 levels differed: while Klein et al. reported the highest IL-6 in vegetarians and the weakest in omnivores [110], our study found higher IL-6 in PESCA and OMN, and lower levels in VEGAN and VEGE, with VEGAN showing the lowest overall.

Two additional studies comparing TNF-α and IL-6 across dietary patterns support our findings. A 2023 study [111] reported elevated TNF-α in vegetarians and vegans compared to omnivores, with vegetarians showing the highest levels. Our data similarly show higher TNF-α in VEGE and VEGAN, and lowest in OMN. Regarding IL-6, both studies and our results indicate the weakest concentrations in VEGAN and the highest in OMN or PESCA.

Another study [112] comparing vegetarians and non-vegetarians found no significant differences in TNF-α levels, but slightly higher IL-6 levels in vegetarians. A separate survey by Franco-de-Moraes [113] reported the lowest TNF-α levels in strict vegetarians, although the differences were not statistically significant.

In our study, hsCRP levels were lower in VEGE and VEGAN compared to PESCA and OMN, although not significantly so. This trend is consistent with international data showing higher hsCRP in omnivores [114,115,116,117,118].

### 4.4. The Relation of the Behavioral Factors of VEGAN to Biochemical Blood Tests

In the VEGAN group, individuals with appropriate behavioral factors (ABF) demonstrated significant positive correlations between key lipid biomarkers—ApoA1, ApoB, and non-HDL-C—and body composition parameters.

#### 4.4.1. ApoA1

ApoA1 levels were positively correlated with body mass, percentage body fat (PBF), and visceral adipose tissue (VAT). As the primary apolipoprotein of HDL-C, ApoA1 is recognized for its anti-atherogenic properties, indicating a protective role in cardiovascular health. However, the observed correlation with increased fat mass may seem paradoxical. ApoA1 can explain this in very-low-density lipoproteins (VLDL) and chylomicrons, which may elevate ApoA1 levels even in individuals with higher adiposity [64,119].

#### 4.4.2. ApoB

ApoB showed strong positive correlations with body mass, BMI, and waist–hip ratio (WHR), indicating that central adiposity is associated with elevated levels of atherogenic lipoproteins. This is particularly relevant for individuals who adhere to a vegan diet and have otherwise healthy behaviors. ApoB is a well-established marker of atherosclerotic risk, and its association with abdominal obesity underscores the importance of evaluating fat distribution, not just total fat mass [120,121].

#### 4.4.3. Non-HDL-C

Non-HDL-C was positively correlated with BFM, PBF, and VAT, suggesting that increased adiposity may influence all cholesterol fractions except HDL-C. Non-HDL-C is considered one of the three primary predictors of cardiovascular risk, alongside LDL-C and ApoB [64,122,123,124]. These findings emphasize that even individuals following an anti-inflammatory vegan diet may be at risk for dyslipidemia if they accumulate excess fat, particularly in the abdominal region.

Therefore, maintaining normal body weight and metabolic balance is essential for cardiovascular protection, regardless of dietary pattern. This is especially important given the role of visceral adipose tissue in promoting chronic low-grade inflammation [125,126].

#### 4.4.4. IL-6

Significant positive correlations were also observed between IL-6 and body mass and WHR, indicating that abdominal fat distribution contributes to elevated levels of this pro-inflammatory cytokine. IL-6 is implicated in the development of insulin resistance, atherosclerosis, and other metabolic disorders [127,128,129].

Although vegan diets are generally considered anti-inflammatory, excess body fat—particularly visceral fat—can activate inflammatory pathways independently of dietary composition [125,130,131]. These findings reinforce the importance of body composition management as a complementary strategy to nutritional interventions in reducing inflammation and cardiometabolic risk.

### 4.5. Integrative Perspective and Broader Implications

Our findings align with previous evidence supporting plant-based diets as effective strategies for reducing cardiometabolic risk [132]. These benefits are consistent with large-scale analyses of Mediterranean and anti-inflammatory dietary patterns. Moreover, mechanistic insights into chronic disorders emphasize the role of immune-inflammatory pathways and metabolic dysregulation in disease progression. As highlighted by [22] in a systems biology approach to anemia of chronic disorders, modeling complex interactions can improve our understanding of pathophysiology. Additionally, the editorial underscores the importance of integrating nutritional biochemistry with personalized interventions to restore homeostasis and reduce disease burden [133]. These perspectives are further supported by previous research conducted by Kwiatkowska and Formanowicz, which demonstrated significant associations between dietary habits and body composition among vegans, vegetarians, pescatarians, and omnivores [47]. The same authors [134] found that individuals on vegetarian (including vegan) diets demonstrate healthier behaviors, particularly in terms of nutrition, compared to those on traditional diets, even during the pandemic. Collectively, these findings underscore the importance of combining high-quality plant-based diets with lifestyle modifications and advanced modeling approaches to optimize cardiovascular and systemic health outcomes.

## 5. Strengths and Limitations of the Study

### 5.1. Strengths

This study offers several notable strengths that enhance the validity and relevance of its findings:

Comprehensive assessment of dietary and behavioral factors: By integrating dietary patterns with lifestyle behaviors (physical activity, sleep, smoking, alcohol consumption), the study provides a multidimensional view of cardiometabolic and inflammatory risk, which is often overlooked in nutrition research.

Detailed subgroup analysis: Stratification into ABF and IBF subgroups within each dietary group—particularly the VEGAN group—allowed for nuanced comparisons and identification of lifestyle-dependent effects, thereby strengthening the interpretability of the results.

Use of advanced biochemical markers: The inclusion of apolipoproteins (ApoA1, ApoB), lipoprotein(a), and inflammatory cytokines (IL-6, TNF-α, hsCRP) provides a deeper insight into cardiovascular and inflammatory status beyond standard lipid panels.

Focus on a young, healthy population: Studying individuals aged 18–39 minimizes confounding from age-related metabolic changes and chronic disease, allowing clearer attribution of outcomes to diet and lifestyle.

Rigorous exclusion criteria were applied, excluding participants with obesity or abnormal laboratory values. This ensured that the sample represented metabolically healthy individuals and reduced bias in biochemical comparisons.

Application of validated tools: Body composition was assessed using DSM-BIA technology, and dietary habits were verified using a structured FFQ, enhancing the reliability of the data.

Novel correlations identified: The study revealed significant associations between lipid and inflammatory markers and body composition metrics, particularly in the VEGAN-ABF subgroup, contributing new evidence to the field of lifestyle medicine.

### 5.2. Limitations

This study has several limitations that should be acknowledged.

First, the cross-sectional design precludes any causal inferences regarding the relationships between dietary patterns, lifestyle behaviors, and biochemical outcomes. Longitudinal or interventional studies would be necessary to confirm the observed associations.

Second, although the sample size was sufficient for subgroup analysis within the VEGAN group, the smaller number of participants in the PESCA and OMN groups may have limited statistical power to detect subtle differences in these populations.

Moreover, the overall sample size was relatively small, which inherently limits the statistical power of the analyses and the ability to perform multivariable adjustments to control for potential confounders. This may increase the risk of both type I and type II errors. The sample size was determined by the availability of eligible participants, and no a priori power calculation was conducted, as the study was designed as an exploratory investigation. Subclassification into appropriate and inappropriate behavioral factor (ABF/IBF) subgroups was conducted for all dietary categories. However, the limited sample sizes in the vegetarian, pescatarian, and omnivore groups precluded the possibility of conducting comprehensive statistical analyses within these subgroups.

Third, self-reported dietary adherence and behavioral factors may be subject to recall or social desirability bias, despite verification through the Food Frequency Questionnaire (FFQ). Objective measures of physical activity and sleep quality could enhance the reliability of behavioral assessments.

Fourth, the study did not include detailed data on supplementation, particularly regarding omega-3 fatty acids, vitamin B12, vitamin D, and calcium, which may influence lipid and homocysteine levels. Future research should incorporate supplementation habits to better understand their impact on biochemical markers.

Fifth, while bioelectrical impedance analysis (BIA) is a practical and non-invasive method for assessing body composition, it may be less accurate than dual-energy X-ray absorptiometry (DXA), especially in estimating visceral adipose tissue.

Additional limitations include the lack of assessment of overall diet quality using standardized indices such as the Dietary Inflammatory Index (DII), the Healthy Eating Index (HEI), the Mediterranean Diet Score (MDS), or the Alternative Healthy Eating Index (AHEI), which could provide deeper insights into the nutritional adequacy and potential anti-inflammatory potential of the participants’ diets. The gender imbalance across groups, particularly the predominance of females in the VEGE and PESCA groups, may affect body composition and biochemical outcomes and limit generalizability. The absence of data on gut microbiota restricts the mechanistic interpretation of inflammatory marker differences. Furthermore, the study did not analyze the impact of the duration of dietary adherence on biochemical outcomes.

Lastly, hormonal status, including menstrual cycle phase, use of hormonal contraception, or menopausal status, was not assessed, which may influence lipid and inflammatory markers, especially in female participants. Moreover, sociodemographic data such as education level, income, occupation, and place of residence were not collected, which limits the ability to account for their potential influence on lifestyle behaviors and biochemical outcomes. Future studies with larger and more balanced samples are needed to validate these results and allow for more robust adjustment for confounding factors.

The study participants were relatively young and predominantly women, representing a population with inherently low cardiovascular risk. Consequently, the observed differences in lipid profiles and inflammatory markers should be interpreted as surrogate indicators rather than direct predictors of atherosclerotic disease. To strengthen the generalizability of these findings, studies involving substantially larger cohorts are needed. In particular, future research involving older individuals or patients with established cardiovascular conditions is warranted to confirm these findings in higher-risk populations.

Despite these limitations, the study offers valuable insights into the interplay between diet, lifestyle, and cardiometabolic health in young adults who adhere to various dietary patterns.

## 6. Conclusions

The VEGAN group demonstrated the most consistent health-promoting behavioral patterns and favorable body composition outcomes. Specifically, this group exhibited the lowest levels of total and visceral adipose tissue, while maintaining the second-highest values for muscle mass, protein content, body water, and mineral levels—comparable to those observed in omnivores, yet distinct from the VEGE and PESCA groups.

Significant intergroup differences were observed for TC, HDL-C, and TG. Subgroup analysis revealed that individuals in the VEGAN-IBF subgroup had significantly higher concentrations of Lp(a), LDL-C, and non-HDL-C, indicating that unhealthy lifestyle behaviors may attenuate the cardiometabolic benefits of a vegan diet.

Moreover, the VEGAN-IBF subgroup exhibited elevated homocysteine levels, supporting the hypothesis that low physical activity and potential vitamin B deficiencies can increase cardiovascular risk, even within plant-based dietary patterns.

Correlation analyses further demonstrated that increased adiposity, particularly visceral fat, was associated with unfavorable lipid and inflammatory profiles, including elevated ApoB, non-HDL-C, and IL-6. These findings suggest that body composition, particularly abdominal fat distribution, plays a crucial role in modulating cardiovascular and inflammatory risk, regardless of dietary adherence.

In summary, while a balanced vegan diet offers substantial health benefits, these advantages are further enhanced by adopting appropriate lifestyle behaviors. Maintaining optimal body composition, particularly by minimizing visceral adiposity and supporting muscle mass, is essential for reducing cardiometabolic and inflammatory risk. These results underscore the importance of integrating dietary quality with physical conditioning in preventive health strategies.

## Figures and Tables

**Table 1:** Lifestyle Characteristics of Participants According to Dietary Group.

Studied Groups	Behavioral Factors Characteristics											
	Level of Activity [min/week]			Sleep Duration [h/night]			Smoking			Vodka Consumption		
	Low (<150)	Medium (150–300)	High (>300)	≤6	7–8	≥9	Never	Occasionally	Regularly	Never	Rare	Monthly/Weekly/Daily
points *	0	0.5	1	0	1	0.5	1	0.5	0	1	0.5	0
	*n* (%)											
VEGAN *n* = 48 F/M: 32/16	9 (18.8)	31 (64.6)	8 (16.7)	3 (6.3)	38 (79.2)	7 (14.6)	40 (83.3)	3 (6.3)	5 (10.5)	35 (72.9)	10 (20.8)	3 (6.3)
VEGE *n* = 49 F/M: 45/4	7 (14.3)	35 (71.4)	7 (14.3)	13 (26.5)	32 (65.3)	4 (8.2)	38 (77.5)	7 (14.3)	4 (8.2)	30 (61.2)	14 (28.6)	5 (10.2)
PESCA *n* = 23 F/M: 20/3	0 (0.0)	19 (82.6)	4 (17.4)	3 (13.0)	20 (87.0)	0 (0.0)	15 (65.2)	4 (17.4)	4 (17.4)	14 (60.9)	4 (17.4)	5 (21.7)
OMN *n* = 35 F/M: 21/14	4 (11.4)	21 (60)	10 (28.6)	7 (20)	27 (77.1)	1 (2.9)	29 (82.9)	1 (2.9)	5 (14.3)	17 (48.6)	14 (40)	4 (11.4)

* points—for a description of awarding points, see Section 2.5. The numerical value assigned to each answer was presented, based on which subgroups were created in each studied group. Abbreviations: F-female; M-male.

**Table 2:** Distribution of Participants into Behavioral Subgroups Based on Lifestyle Score.

Subgroups	VEGAN	VEGE	PESCA	OMN
	*n* (%)			
**ABF** **(>3 points)**	25 (52.08)	16 (32.65)	9 (39.13)	11 (31.43)
**IBF** **(≤3 points)**	23 (47.92)	33 (67.35)	14 (60.87)	24 (68.57)

Abbreviations: ABF—Appropriate Behavioral Factors; IBF—Inappropriate Behavioral Factors.

**Table 3:** Body composition characteristics in the studied groups.

Studied Groups	*n*	Body Composition Parameters Mean ± SD									
		Body Water [l]	Protein [kg]	Minerals [kg]	BFM [kg]	Body Mass [kg]	BMI [kg/m^2^]	PBF [%]	SMM [kg]	WHR	VAT [cm^2^]
VEGAN	48	36.0 ± 6.78	9.66 ± 1.85	3.41 ± 0.576	13.3 ± 4.77	62.4 ± 9.48	21.0 ± 2.24	21.4 ± 7.22	27.2 ± 5.59	0.831 ± 0.041	5.27 ± 2.39
VEGE	49	32.4 ± 4.57	8.68 ± 1.25	3.14 ± 0.432	14.4 ± 3.48	58.7 ± 7.62	20.9 ± 1.96	24.6 ± 7.22	24.2 ± 3.77	0.824 ± 0.038	5.57 ± 1.65
PESCA	23	33.1 ± 5.47	8.85 ± 1.51	3.19 ± 0.512	14.1 ± 4.91	59.2 ± 10.5	20.8 ± 2.24	23.4 ± 5.94	24.7 ± 4.57	0.832 ± 0.042	5.52 ± 2.21
OMN	35	37.4 ± 8.71	10.1 ± 2.38	3.53 ± 0.742	14.7 ± 5.05	65.7 ± 14.4	21.9 ± 3.10	22.4 ± 6.00	28.4 ± 7.19	0.846 ± 0.044	5.66 ± 2.41
*p*-value *		0.016	0.015	0.033	0.404	0.088	0.694	0.065	0.014	0.281	0.713

* *p* was calculated to evaluate differences between the groups, including all participants regardless of gender. Analyses were then conducted to examine differences between genders, but no significant differences were found. Abbreviations: BFM—Body Fat Mass; BMI—Body Mass Index; PBF—Percentage Body Fat; SMM—Skeletal Muscle Mass; WHR—Waist-Hip Ratio; VAT—Visceral Adipose Tissue.

**Table 4:** Lipid Profile Parameters Across Dietary Groups.

Studied Groups and Subgroups		*n*	Lipid Profile Mean ± SD									
			ApoA1 [mg/mL]	ApoB [mg/mL]	ApoB/ ApoA1	Lp(a) [mg/dL]	TC [mg/dL]	HDL-C [mg/dL]	LDL-C [mg/dL]	TG [mg/dL]	Non-HDL-C [mg/dL]	TC/ HDL-C
VEGAN	All	48	1.80 ± 1.87	1.15 ± 0.983	0.900 ± 1.06	22.5 ± 17.6	142 ± 29.2	51.7 ± 10.1	70.0 ± 21.0	95.1 ± 26.4	90.1 ± 25.9	2.79 ± 0.596
VEGE	All	49	1.30 ± 0.948	1.04 ± 0.700	0.944 ± 0.684	23.6 ± 17.4	157 ± 22.8	59.3 ± 13.4	74.1 ± 18.2	94.0 ± 27.2	98.1 ± 19.9	2.76 ± 0.613
PESCA	All	23	1.56 ± 1.09	0.957 ± 0.495	0.871 ± 0.811	19.1 ± 16.1	160 ± 34.7	60.6 ± 13.0	75.1 ± 24.8	92.4 ± 45.9	99.6 ± 35.0	2.73 ± 0.698
OMN	All	35	2.14 ± 1.77	1.05 ± 0.744	0.914 ± 1.28	23.8 ± 18.0	158 ± 30.3	55.8 ± 14.3	80.1 ± 20.9	77.9 ± 34.7	102 ± 27.9	2.94 ± 0.778
*p*-value ^(1)^*			0.380	0.983	0.107	0.404	0.032	0.006	0.133	0.006	0.297	0.746
All participants	ABF	94	1.61 ± 1.454	1.01 ± 0.663	1.00 ± 1.149	19.46 ± 12.956	149 ± 30.898	56.08 ± 13.817	71.32 ± 20.285	91.21 ± 36.109	93.90 ± 27.260	2.78 ± 0.702
	IBF	61	1.799 ± 1.625	1.139 ± 0.935	0.773 ± 0.593	27.493 ± 21.659	157 ± 26.080	56.770 ± 11.694	79.046 ± 21.085	89.098 ± 26.414	100.967 ± 24.647	2.858 ± 0.587
*p*-value ^(2)^*			0.311 ^(U)^	0.965 ^(U)^	0.335 ^(U)^	0.042 ^(U)^	0.107 ^(tS)^	0.554 ^(U)^	0.024 ^(tS)^	0.812 ^(U)^	0.104 ^(tS)^	0.188 ^(U)^

* *p* ^(1)^ was calculated to assess differences between the groups; * *p* ^(2)^ was calculated to assess differences between ABF and IBF in all participants concerning selected variables; tS—Student’s *t*-test; U—Mann–Whitney U test. Abbreviations: ABF—Appropriate Behavioral Factors; ApoA1—Apolipoprotein A1; ApoB—Apolipoprotein B; ApoB/ApoA1—apolipoprotein B/apolipoprotein A1 ratio; HDL-C—High-density lipoprotein cholesterol; IBF—Inappropriate Behavioral Factors; LDL-C—Low-density lipoprotein cholesterol; Lp(a)—Lipoprotein(a); Non-HDL-C—Non-high-density lipoprotein cholesterol; TC—Total cholesterol; TC/HDL-C—Total cholesterol to high-density lipoprotein cholesterol ratio; TG—triacylglycerols.

**Table 5:** Lipid Profile Parameters Across Dietary Groups with female and male divisions.

Female and Male Division		*n*	Lipid Profile Mean ± SD		
			ApoA1 [mg/mL]	ApoB/ ApoA1	HDL-C [mg/dL]
VEGAN	F	32	1.92 ± 2.01	0.805 ± 0.669	53.4 ± 10.6
	M	16	1.55 ± 1.59	1.09 ± 1.60	48.3 ± 8.43
VEGE	F	45	1.33 ± 0.980	0.975 ± 0.702	60.7 ± 12.9
	M	4	0.946 ± 0.317	0.600 ± 0.290	43.8 ± 6.99
PESC	F	20	1.64 ± 1.15	0.873 ± 0.859	61.9 ± 13.0
	M	3	1.05 ± 0.151	0.852 ± 0.461	52.0 ± 11.0
OMN	F	21	2.17 ± 1.78	0.763 ± 0.680	60.8 ± 15.6
	M	14	2.09 ± 1.83	1.14 ± 1.87	48.4 ± 8.04
*p*-value for F			0.561	0.172	0.055
*p*-value for M			0.608	0.820	0.607

*p* was calculated to assess differences between groups, including separately for gender. Abbreviations: ApoA1—Apolipoprotein A1; ApoB/ApoA1—apolipoprotein B/apolipoprotein A1 ratio; HDL-C—High-density lipoprotein cholesterol; F—female; M—male.

**Table 6:** Lipid Profile Parameters in VEGAN Subgroups According to Behavioral Factors.

VEGAN Subgroups	*n*	Lipids Profile Mean ± SD									
		ApoA1 [mg/mL]	ApoB [mg/mL]	ApoB/ ApoA1	Lp(a) [mg/dL]	TC [mg/dL]	HDL-C [mg/dL]	LDL-C [mg/dL]	TG [mg/dL]	Non-HDL-C [mg/dL]	TC/ HDL-C
ABF	23	1.308 ± 1.361	0.777 ± 0.484	0.956 ± 1.347	17.061 ± 10.064	133.435 ± 32.020	51.287 ± 10.160	61.322 ± 20.617	99.652 ± 32.730	82.148 ± 27.410	2.659 ± 0.575
IBF	25	2.250 ± 2.173	1.499 ± 1.191	0.846 ± 0.7333	27.457 ± 21.449	149.560 ± 24.607	52.080 ± 10.243	78.048 ± 18.308	90.480 ± 18.948	97.480 ± 22.600	2.942 ± 0.585
*p*-value *		0.110 ^(U)^	0.050 ^(U)^	0.902 ^(U)^	0.029 ^(U)^	0.055 ^(tS)^	0.789 ^(tS)^	0.005 ^(tS)^	0.248 ^(tW)^	0.039 ^(tS)^	0.110 ^(U)^

* *p* was calculated to assess differences between ABF and IBF in the VEGAN group; tS—Student’s *t*-test; tW—Welch’s *t*-test; U—Mann–Whitney U test. Abbreviations: ABF—Appropriate Behavioral Factors; ApoA1—Apolipoprotein A1; ApoB—Apolipoprotein B; ApoB/ApoA1—apolipoprotein B/apolipoprotein A1 ratio; HDL-C—High-density lipoprotein cholesterol; IBF—Inappropriate Behavioral Factors; LDL-C—Low-density lipoprotein cholesterol; Lp(a)—Lipoprotein(a); Non-HDL-C—Non-high-density lipoprotein cholesterol; TC—Total cholesterol; TC/HDL-C—Total cholesterol to high-density lipoprotein cholesterol ratio; TG—triacylglycerols.

**Table 7:** Metabolic and Inflammatory Marker Levels Across Dietary and Behavioral Subgroups.

Studied Groups and Subgroups		*n*	Metabolic and Inflammatory Markers Mean ± SD			
			TNF-α [ng/L]	HCY [µmol/L]	IL-6 [pg/mL]	hsCRP [mg/L]
VEGAN	All	48	128 ± 90.6	14.3 ± 10.9	2.79 ± 2.27	4.76 ± 3.07
VEGE	All	49	121 ± 83.8	12.8 ± 8.17	2.87 ± 2.31	3.83 ± 2.50
PESCA	All	23	111 ± 49.1	15.3 ± 8.78	3.16 ± 2.40	5.47 ± 3.45
OMN	All	35	89.1 ± 39.2	10.0 ± 5.53	3.05 ± 1.87	5.19 ± 3.01
*p*-value ^(1)^*			0.149	0.096	0.833	0.100
All participants	ABF	94	111.18 ± 64.12	12.29 ± 7.84	3.15 ± 2.16	4.75 ± 3.08
	IBF	61	119.282 ± 89.12	14.166 ± 10.13	2.601 ± 2.25	4.540 ± 2.85
*p*-value ^(2)^*			0.281	0.714	0.076	0.815
VEGAN	ABF	23	93.403 ± 37.8	9.914 ± 6.41	3.171 ± 2.28	5.305 ± 3.15
	IBF	25	159.909 ± 112.0	18.371 ± 12.57	2.444 ± 2.26	4.260 ± 2.97
*p*-value ^(3)^*			0.110	0.007	0.122	0.187

* *p* ^(1)^ was calculated to assess differences between groups concerning selected variables. * *p* ^(2)^ was calculated to assess differences between ABF and IBF in all participants (Mann–Whitney U test). * *p* ^(3)^ was calculated to assess differences between ABF and IBF in the VEGAN group (Mann–Whitney U test). Abbreviations: ABF—Appropriate Behavioral Factors; HCY—Homocysteine; hsCRP—High-sensitivity C-reactive protein; IBF—Inappropriate Behavioral Factors; IL-6—Interleukin 6; TNF-α—Tumor Necrosis Factor-alpha.

**Table 8:** Significant correlations between biochemical parameters and body composition metrics in the VEGAN-ABF subgroup.

Blood Lipids and Lipoproteins	Body Composition Parameter		
**ApoA1** [mg/dL]	**BFM [kg]**	**PBF [%]**	**VAT [cm^2^]**
	0.452 (ρ) *p* < 0.05	0.476 (ρ) *p* < 0.05	0.433 (ρ) *p* < 0.05
**ApoB** [mg/dL]	**Body mass [kg]**	**BMI [kg/m^2^]**	**WHR**
	0.461 (ρ) *p* < 0.05	0.517 (ρ) *p* < 0.05	0.489 (ρ) *p* < 0.05
**Non-HDL-C** [mg/dL]	**BFM [kg]**	**PBF [%]**	**VAT [cm^2^]**
	0.432 (r) *p* < 0.05	0.42 (r) *p* < 0.05	0.461 (r) *p* < 0.05
**Metabolic and inflammatory markers**			
**IL-6** [pg/mL]	**Body mass [kg]**	**WHR**	
	0.526 (ρ) *p* < 0.01	0.499 (ρ) *p* < 0.05	

ρ—Spearman correlation; r—Pearson correlation. Abbreviations: ApoA1—Apolipoprotein A1; ApoB—Apolipoprotein B; PBF—Percentage Body Fat; BFM—Body Fat Mass; BMI—Body Mass Index; IL-6—Interleukin 6; Non-HDL-C—Non-high-density lipoprotein cholesterol; WHR—Waist-Hip Ratio; VAT—Visceral Adipose Tissue.

## Data Availability

The original contributions presented in this study are included in the article and Supplementary Materials. Further inquiries can be directed to the corresponding author.

## Supplementary Materials

The following supporting information can be downloaded at https://www.mdpi.com/article/10.3390/nu17233634/s1, Table S1: Biochemical methods and reference ranges; Table S2: DSCF pairwise comparisons for significant body composition parameters; Table S3: DSCF pairwise comparisons for significant lipid profile parameters. References [135,136,137,138] are cited in Supplementary Materials.

## Abbreviations

The following abbreviations are used in this manuscript:

ABF	Appropriate Behavioral Factors
ApoA1	Apolipoprotein A1
ApoB	Apolipoprotein B
ApoB/ApoA1	ApoB to ApoA1 ratio
BFM	Body Fat Mass
BIA	Bioelectric Impedance
BMI	Body Mass Index
CI	Confidence Intervals
CRP	C-reactive protein
DASH	Dietary Approaches to Stop Hypertension
DII	Dietary Inflammatory Index
DSM-BIA	Direct Segmental Multi-frequency Bioelectrical Impedance Analysis
DXA	Dual-Energy X-ray Absorptiometry
EAS	European Atherosclerosis Society
ELISA	Immunoenzymatic Enzyme-Linked Immunosorbent Assay
ESC	European Society of Cardiology
F	Female
FFQ	Food Frequency Questionnaire
GLU	Glucose Levels
HCY	Homocysteine
HDL-C	High-Density Lipoprotein Cholesterol
hsCRP	High-Sensitivity C-Reactive Protein
IBF	Inappropriate Behavioral Factors
IL-6	Interleukin 6
LDL-C	Low-Density Lipoprotein Cholesterol
Lp(a)	Lipoprotein(a)
M	Male
MUFAs	Monounsaturated Fatty Acids
NCDs	Non-Communicable Diseases
OMN	Omnivores
PBF	Percentage Body Fat
PESCA	Pescatarian Diet
PPBD	Predominantly Plant-Based Diets
PUFAs	Polyunsaturated Fatty Acids
r	Pearson Correlation
SMM	Skeletal Muscle Mass
TC	Total Cholesterol
TG	Triacylglycerols
TNF-α	Tumor Necrosis Factor-alpha
tS	Student’s *t*-test
tW	Welch’s *t*-test
U	Mann–Whitney U test
VAT	Visceral Adipose Tissue
VEGAN	Vegan Diet
VEGE	Lacto-/Ovo-Vegetarian Diet
WHO	Health Organization
WHR	Waist-Hip Ratio
ρ	Spearman Correlation
