# Supplementary material for: Dietary and Lifestyle Patterns and Their Associations with Cardiovascular and Inflammatory Biomarkers in Vegans, Vegetarians, Pescatarians, and Omnivores: A Cross-Sectional Study
Source: Nutrients. 2025 Nov 21;17(23):3634. doi: 10.3390/nu17233634 (PMC12694547; doi:10.3390/nu17233634)
Supplement: Supplementary file 1 [file nutrients-17-03634-s001.zip › Table S1.pdf]

Supplementary Materials:

TABLE S1: Biochemical methods and reference ranges.

Parameter	Method/Instrument	95% confidence interval (CI)	Reference range	Reference
**ApoA1 [mg/ml]**	Enzyme-linked Immunosorbent Assay (ELISA)	OMN: CI **1.55-2.73**<br>PESCA: CI **1.12-2.01**<br>VEGAN: CI **1.27-2.33**<br>VEGE: CI **1.03-1.57**	**-**	-
**ApoB [mg/ml]**	Enzyme-linked Immunosorbent Assay (ELISA)	OMN: CI **0.800-1.29**<br>PESCA: CI **0.755-1.16**<br>VEGAN: CI **0.875-1.43**<br>VEGE: CI **0.841-1.23**	**< 100mg/dL**	ESC Guidelines on Cardiovascular Disease Prevention in Clinical Practice. Eur Heart J 2021, 42, 3227–3337,
**ApoB/ApoA1**	Calculated	OMN: CI **0.490-1.34**<br>PESCA: CI **0.539-1.20**<br>VEGAN: CI **0.600-1.20**<br>VEGE: CI **0.752-1.14**	**-**	-
**Lp(a) [mg/dL]**	Enzyme-linked Immunosorbent Assay (ELISA)	OMN: CI **17.09-29.8**<br>PESCA: CI **12.5-25.7**<br>VEGAN: CI **17.5-27.5**<br>VEGE: CI **18.7-28.4**	**< 50**	Guideline on the Management of Blood Cholesterol. Journal of the American College of Cardiology 2019, 73, e285–e350, doi:10.1016/j.jacc.2018.11.003.
**TC [mg/dL]**	Cobas b101, Roche	-	**< 190**	ESC Guidelines on Cardiovascular Disease Prevention in Clinical Practice. Eur Heart J 2021, 42, 3227–3337,
**HDL-C [mg/dL]**	Cobas b101, Roche	-	**Female: > 50 mg/dl**<br>**Male: > 40 mg/dl**	Guideline on the Management of Blood Cholesterol. Journal of the American College of Cardiology 2019, 73, e285–e350, doi:10.1016/j.jacc.2018.11.003.
**LDL-C [mg/dL]**	Cobas b101, Roche	-	**< 116**	ESC Guidelines on Cardiovascular Disease Prevention in Clinical Practice. Eur Heart J 2021, 42, 3227–3337,
**TG [mg/dL]**	Cobas b101, Roche	-	**< 150**	ESC Guidelines on Cardiovascular Disease Prevention in Clinical Practice. Eur Heart J 2021, 42, 3227–3337,
**Non-HDL**	Calculated	-	**< 130**	ESC Guidelines on Cardiovascular Disease Prevention in Clinical Practice. Eur Heart J 2021, 42, 3227–3337
**TC/HDL cholesterol**	Calculated	-	**< 6**	https://www.nhs.uk/conditions/high-cholesterol/cholesterol-levels/
**HCY [μmol/L]**	Enzyme-linked Immunosorbent Assay (ELISA)	OMN: CI **8.22-11.9**<br>PESCA: CI **11.7-18.8**<br>VEGAN: CI **11.2-17.4**<br>VEGE: CI **10.5-15.1**	**< 15**	Yang B, Fan S, Zhi X, et al. Prevalence of hyperhomocysteinemia in China: a systematic review and meta-analysis. Nutrients 2014;7:74–90.<br><br>Wang C, Wu Q, Zhang L, et al. Elevated total plasma homocysteine levels are associated with type 2 diabetes in women with hypertension. Asia Pacific J Clin Nutr 2015;24:683–91.<br><br>Feng, Y.; Kang, K.; Xue, Q.; Chen, Y.; Wang, W.; Cao, J. Value of Plasma Homocysteine to Predict Stroke, Cardiovascular Diseases, and New-Onset Hypertension: A Retrospective

				Cohort Study. Medicine 2020, 99, e21541, doi:10.1097/MD.0000000000021541.
**TNF-alpha [ng/l]**	Enzyme-linked Immunosorbent Assay (ELISA)	OMN: CI **76.2-102**<br>PESCA: CI **90.4-131**<br>VEGAN: CI **102-154**<br>VEGE: CI **97.4-144**	-	-
**hsCRP [mg/L]**	Enzyme-linked Immunosorbent Assay (ELISA)	OMN: CI **4.19-6.18**<br>PESCA: CI **4.06-6.88**<br>VEGAN: CI **3.89-5.63**<br>VEGE: CI **3.13-4.53**	**< 3**	Pearson T.A., Mensah G.A., Alexander R.W. i wsp. Cen- ters for Disease Control and Prevention; American Heart As- sociation. Markers of inflammation and cardiovascular dise- ase: application to clinical and public health practice: A state- ment for healthcare professionals from the Centers for Disease Control and Prevention and the American Heart Association. Circulation 2003; 107: 499–511.
**IL-6 [pg/ml]**	Enzyme-linked Immunosorbent Assay (ELISA)	OMN: CI **2.43-3.67**<br>PESCA: CI **2.8-4.14**<br>VEGAN: CI **2.15-3.44**<br>VEGE: CI **2.23-3.52**	-	-
