# Supplementary material for: Dietary and Lifestyle Patterns and Their Associations with Cardiovascular and Inflammatory Biomarkers in Vegans, Vegetarians, Pescatarians, and Omnivores: A Cross-Sectional Study
Source: Nutrients. 2025 Nov 21;17(23):3634. doi: 10.3390/nu17233634 (PMC12694547; doi:10.3390/nu17233634)
Supplement: Supplementary file 1 [file nutrients-17-03634-s001.zip › Table S2.pdf]

Supplementary Materials:

TABLE S2: DSCF pairwise comparisons for significant body composition parameters.

**Body composition parameter**	**Comparison**	***p*-value**
Body water	OMN vs PESCA	0.495
Body water	OMN vs VEGAN	0.994
Body water	OMN vs VEGE	0.119
Body water	PESCA vs VEGAN	0.251
Body water	PESCA vs VEGE	0.948
Body water	VEGAN vs VEGE	0.019
Protein	OMN vs PESCA	0.450
Protein	OMN vs VEGAN	0.991
Protein	OMN vs VEGE	0.101
Protein	PESCA vs VEGAN	0.245
Protein	PESCA vs VEGE	0.977
Protein	VEGAN vs VEGE	0.022
Minerals	OMN vs PESCA	0.480
Minerals	OMN vs VEGAN	0.971
Minerals	OMN vs VEGE	0.135
Minerals	PESCA vs VEGAN	0.405
Minerals	PESCA vs VEGE	0.967
Minerals	VEGAN vs VEGE	0.048
Skeletal Muscle Mass	OMN vs PESCA	0.436
Skeletal Muscle Mass	OMN vs VEGAN	0.990
Skeletal Muscle Mass	OMN vs VEGE	0.101
Skeletal Muscle Mass	PESCA vs VEGAN	0.251
Skeletal Muscle Mass	PESCA vs VEGE	0.970
Skeletal Muscle Mass	VEGAN vs VEGE	0.020
