# Supplementary material for: Dietary and Lifestyle Patterns and Their Associations with Cardiovascular and Inflammatory Biomarkers in Vegans, Vegetarians, Pescatarians, and Omnivores: A Cross-Sectional Study
Source: Nutrients. 2025 Nov 21;17(23):3634. doi: 10.3390/nu17233634 (PMC12694547; doi:10.3390/nu17233634)
Supplement: Supplementary file 1 [file nutrients-17-03634-s001.zip › Table S3.pdf]

**Supplementary Materials:**

**TABLE S3**: DSCF pairwise comparisons for significant lipid profile parameters.

| **Lipid profile** | **Comparison** | ***p*-value** |
|---|---|---|
| Total cholesterol | OMN vs PESCA | 0.826 |
| Total cholesterol | OMN vs VEGAN | 0.752 |
| Total cholesterol | OMN vs VEGE | 0.284 |
| Total cholesterol | PESCA vs VEGAN | 0.998 |
| Total cholesterol | PESCA vs VEGE | 0.885 |
| Total cholesterol | VEGAN vs VEGE | 0.907 |
| High-density lipoprotein cholesterol | OMN vs PESCA | 0.362 |
| High-density lipoprotein cholesterol | OMN vs VEGAN | 0.702 |
| High-density lipoprotein cholesterol | OMN vs VEGE | 0.339 |
| High-density lipoprotein cholesterol | PESCA vs VEGAN | 0.024 |
| High-density lipoprotein cholesterol | PESCA vs VEGE | 1.000 |
| High-density lipoprotein cholesterol | VEGAN vs VEGE | 0.015 |
| Triacylglycerols | OMN vs PESCA | 0.706 |
| Triacylglycerols | OMN vs VEGAN | 0.007 |
| Triacylglycerols | OMN vs VEGE | 0.017 |
| Triacylglycerols | PESCA vs VEGAN | 0.503 |
| Triacylglycerols | PESCA vs VEGE | 0.660 |
| Triacylglycerols | VEGAN vs VEGE | 0.993 |
